# Second pilot trials of the STAR-Liege protocol for tight glycemic control in critically ill patients

**DOI:** 10.1186/1475-925X-11-58

**Published:** 2012-08-23

**Authors:** Sophie Penning, Aaron J Le Compte, Paul Massion, Katherine T Moorhead, Christopher G Pretty, Jean-Charles Preiser, Geoffrey M Shaw, Fatanah Suhaimi, Thomas Desaive, J Geoffrey Chase

**Affiliations:** 1 Dept of Mechanical Eng, Centre for Bio-Engineering, University of Canterbury, Christchurch,, Private Bag 4800, 8054, New Zealand; 2 Cardiovascular Research Centre, Institut de Physique, Université de Liege, Institut de Physics, Allée du 6 Août, 17 (Bât B5), Liege, B4000 Liege, Liege, Belgium; 3 Dept of Intensive Care, Erasme University Hospital, 808 route de Lennik, Brussels, B1070, Belgium; 4 University of Otago Christchurch, School of Medicine, Christchurch, 8054, New Zealand; 5 Dept of Intensive Care, Christchurch Hospital, Christchurch, 8054, New Zealand; 6 Dept of Intensive Care, CHU, Liege, Belgium

**Keywords:** Glycemic control, Critical care, Intensive care unit, Pilot trial

## Abstract

**Background:**

Critically ill patients often present increased insulin resistance and stress-induced hyperglycemia. Tight glycemic control aims to reduce blood glucose (BG) levels and variability while ensuring safety from hypoglycemia. This paper presents the results of the second Belgian clinical trial using the customizable STAR framework in a target-to-range control approach. The main objective is reducing measurement frequency while maintaining performance and safety of the glycemic control.

**Methods:**

The STAR-Liege 2 (SL2) protocol targeted the 100–140 mg/dL glycemic band and offered 2-hourly and 3-hourly interventions. Only insulin rates were adjusted, and nutrition inputs were left to the attending clinicians. This protocol restricted the forecasted risk of BG < 90 mg/dL to a 5% level using a stochastic model of insulin sensitivity to assess patient-specific responses to insulin and its future likely variability to optimize insulin interventions. The clinical trial was performed at the Centre Hospitalier Universitaire de Liege and included 9 patients. Results are compared to 24-hour pre-trial and 24-hour post-trial, but also to the results of the first pilot trial performed in Liege, STAR-Liege 1 (SL1). This trial was approved by the Ethics Committee of the Medical Faculty of the University of Liege (Liege, Belgium).

**Results:**

During the SL2 trial, 91 measurements were taken over 194 hours. BG levels were tightly distributed: 54.9% of BG within 100–140 mg/dL, 40.7% were ≥ 140 mg/dL and 4.4% were < 100 mg/dL with no BG < 70 mg/dL. Comparing these results with 24-hour pre-trial and post-trial shows that SL2 reduced high and low BG levels and reduced glycemic variability. Nurses selected 3-hourly measurement only 5 of 16 times and overrode 12% of 91 recommended interventions (35% increased insulin rates and 65% decreased insulin rates). SL1 and SL2 present similar BG levels distribution (p > 0.05) with significantly reduced measurement frequency for SL2 (p < 0.05).

**Conclusions:**

The SL2 protocol succeeded in reducing clinical workload while maintaining safety and effectiveness of the glycemic control. SL2 was also shown to be safer and tighter than hospital control. Overall results validate the efficacy of significantly customizing the STAR framework.

## Background

In critically ill patients, increased insulin resistance due to stress results in stress-induced hyperglycemia, which is linked to worsened patient outcomes and increased mortality [1-4]. Tight glycemic control (TGC) seeks to reduce the blood glucose (BG) levels and variability associated with negative outcomes, and can reduce mortality up to 45% [5-7]. However, a high risk of hypoglycemia and inability to repeat successful results have been associated with many TGC protocols [8], where limiting low BG levels is crucial to ensure control safety [3,9,10].

High inter- and intra- patient variability makes successful TGC difficult [11-13]. Model-based controllers use computer models of patient physiology to capture patient-specific response to insulin and nutrition inputs. Thus, they enable TGC protocols to predict patient response to optimize interventions and resulting BG levels in the presence of variability [14-17].

STAR (Stochastic TARgeted) is a flexible model-based control approach that enables adaptive, patient-specific TGC. STAR directly accounts for evolving physiological patient condition and inter- and intra- patient variability by identifying insulin sensitivity (SI) and its future variability [11] at each intervention to optimize control and safety. STAR can be customized for clinically specified glycemic targets, control approaches (e.g. insulin only, insulin and nutrition, *etc.*) and clinical resources (e.g. measurement frequency).

The STAR framework was previously customized in glycemic target and control intervention to match clinical standards at the Centre Hospitalier Universitaire (CHU) of Liege, Belgium [18]. This paper presents a significantly modified and improved STAR-Liege protocol, based on issues highlighted in the first pilot trial results. In particular, only 2 and 3 hourly insulin interventions were offered. The goal was changed to maximize the overlap of the potential (5^th^-95^th^ percentile) glycemic outcome range with a clinically specified 100–140 mg/dL band, a target-to-range approach. In contrast, the prior approach had a specific target (125 mg/dL) and offered 1-hourly interventions that resulted in 20–24 measurements per day. Finally, the stochastic models were updated to better account for the more variable cardiovascular surgery patient cohort in this intensive care unit (ICU), where recent studies found these patients to be more insulin resistant and variable (greater intra-patient variability), especially for the first days of ICU stay than the overall medical ICU cohort as a whole [13]. Hence, the stochastic model used here directly accounted for this variability by using clinical data specific to cardiac-surgery patients and for the first days of stay. The main objective of these second clinical trials was reducing clinical workload for a highly variable ICU cohort, while maintaining control quality and safety, by using a target-to-range approach.

## Method

### STAR-Liege 2 protocol

**Figure 1:**
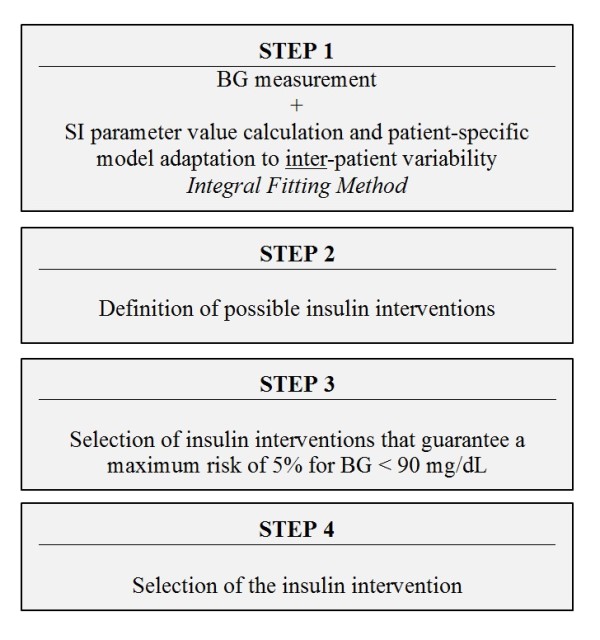
Steps of the STAR-Liege 2 protocol.

Three major changes were made for the STAR-Liege 2 (SL2) protocol. First, the clinically specified glycemic target of 125 mg/dL was changed to a target band (100–140 mg/dL). Second, measurement frequency was reduced, and only 2-hourly and 3-hourly interventions were used to reduce workload. These intervals allowed insulin infusions sufficient time to act so that the controller could more accurately identify insulin action. Finally, the SL2 protocol differed from the SL1 in that it did not specify any nutrition whatsoever. During the SL1 pilot trial, nutrition administration was generally left to the attending clinician who had to enter nutrition rates (and their changes) into the controller to account for the patient feeding. However, the SL1 protocol did recommended increased nutrition rates at low BG concentrations, to prevent unintended hypoglycemia. The SL2 protocol removed these recommendations, making the controller more simple and transparent. An improved glucose-insulin system model was also used (denoted ICING-2 [19]) and described in the Additional file 1. The four main steps of the new SL2 protocol are illustrated in Figure 1. They are described in detail below:

1. Previous and current BG measurements and clinical data (nutrition and insulin rates) are used to identify the current patient-specific model-based SI parameter value for the prior time interval [20]. This step accounts for inter-patient variability by adapting the model estimate of patient response to insulin [12].

2. All possible insulin rates are assessed, based on a minimum insulin rate (0 U/h), a 0.5 U/h insulin rate increment and a maximum insulin rate (*u*_*max*_), of the current insulin rate + 2 U/h up to a maximum of 6 U/h. Note that the insulin rate of 0.5 U/h is not considered due to its small size. Thus, possible insulin rates are 0, 1, 1.5, 2…6 U/h.

However, in two specific cases, no insulin is required. First, when the current BG level is lower than 90 mg/dL; second, when the current BG value is more than 18 mg/dL below the 5^th^ percentile expected from the last controller intervention.

3. For each control interval (2 and 3 hours), the glycemic outcomes of all possible insulin interventions (defined in Step 2) are assessed. Only interventions that predicted a maximum risk of 5% or less for BG < 90 mg/dL, for safety from moderate (< 60 mg/dL) or severe (< 40 mg/dL) hypoglycemia [9], are considered feasible. More precisely, the assessment of each possible insulin intervention includes 3 phases:

a) The stochastic model [11] provides a distribution of possible SI parameter values for the next 2 (or 3) hours, based on the current SI value (identified in Step 1). This phase accounts for the intra-patient variability typically observed in critically ill patients.

b) The 5^th^ and the 50^th^ (median) percentile BG outcome predictions (*BG*_*5th*_ and *BG*_*50th*_ respectively) are calculated using the insulin-glucose system model and using the 95^th^ and 50^th^ (median), respectively, percentile expected SI values obtained from Phase (a). The *BG*_*5th*_ value illustrates the possible BG spread towards hypoglycemia due to intra-patient variability.

c) Hypoglycemic risk is assessed from the 5^th^ percentile BG value to restrict the forecasted risk of BG < 90 mg/dL to a 5% level. Thus *BG*_*5th*_ ≥ 90 mg/dL must be true for the insulin intervention to be feasible. For each time interval (2 and 3 hours), the goal is to find the insulin rates that put the 5th percentile BG closest to the lower bound of the target range (chosen as 100 mg/dL for this study) to maximize overlap of the outcome BG range with the desired target range. Additionally, *BG*_*50th*_ < 140 mg/dL is required for 3-hourly measurements, otherwise, only a 2-hour interval is offered.

4. The insulin intervention associated with the longest feasible time interval for the next BG measurement is selected to minimize workload.

Step 4 was changed for control of Patients 4 to 10 to allow nurses greater freedom. When 3-hourly measurements were available, three options were offered:

a) 2-hourly measurement, and insulin rate forecasted to achieve closest BG to target after 2 hours (*u*_1_)

b) 3-hourly measurement and insulin rate forecasted to achieve closest BG to target after 3 hours (*u*_2_). Only patients 1–3 received this option;

c) 3-hourly measurement and insulin rate using the lesser of the 2-hourly or 3-hourly insulin rates (min(*u*_1_,*u*_2_)).

By default, the controller would have chosen option (b), if available. The change to Step 4 enabled greater nursing flexibility and choice that better reflects STAR framework usage elsewhere [21] and made the system more user friendly.

**Figure 2:**
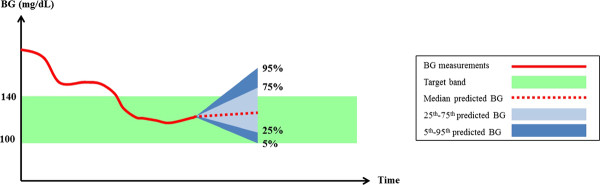
**Overlapping of glycemic target band (green) and stochastic predicted range (blue) with *BG***_***5th***_**≥ 90 mg/dL.**

The SL2 protocol was characterized by two glycemic bands (Figure 2): the 100–140 mg/dL target band and the range of glycemic outcomes due to insulin sensitivity variability (Step 3.b). The controller aimed to maximize the overlap between these two bands, subject to the 5^th^ percentile BG ≥ 90 mg/dL.

### Stochastic model

The stochastic model used with STAR described the hourly changes in insulin sensitivity to improve assessment of the patient’s insulin response (Additional file 2). It was based on clinically observed insulin sensitivity variations in ICU populations [11]. The first pilot STAR-Liege trial (SL1) showed that post-operative cardiac surgery patients were significantly more variable in their insulin sensitivity (SI) than expected during the first post-operative hours [13]. In a final major difference from the first pilot trials in Liege, the stochastic model used in this study directly accounts for this variability by using clinical data specific to cardiac-surgery patients and for the first days of stay. More precisely, it combined the data of cardiac surgery patients from the SPRINT study [5] with the data of all the patients of the Glucontrol study [22], to create a more cohort-specific stochastic model that was unique to this patient group and study.

### Clinical trial and patients

The SL2 protocol was tested in November and December 2010 at the CHU in Liege, Belgium. Each pilot trial was 24 hours long. The clinical trial included 9 patients from the hospital’s intensive care unit, 3 patients (Patients 2, 5 and 9) were in the first 48 hours post-surgery. Initially, patients were recruited if they had two consecutive blood glucose levels > 145 mg/dL. In practice, clinicians also included highly glycemically variable patients (Patients 1, 2 and 5). The clinician stopped patient 2 after 7 hours due to the diagnosis of pancreatic disease. Table 1 shows the patient details and per-patient control information. The Ethics Committee of the Medical Faculty of the University of Liege (Liege, Belgium) granted approval for this trial and the audit, analysis and publication of these data.

**Table 1:** Patient demographic and clinical data relevant to control

		**Patient 1**	**Patient 2**	**Patient 3**	**Patient 4**	**Patient 5**	**Patient 6**	**Patient 7**	**Patient 8**	**Patient 9**
**General information**	**Date of birth**	22/11/1938	9/09/1938	8/12/1959	11/07/1922	11/12/1938	9/09/1938	5/12/1944	19/05/1951	7/01/1939
	**Sex**	F	F	M	M	M	F	M	F	F
	**Diagnosis**	Gastro	Cardio	Trauma	Neurological	Respiratory	Cardio (then pneumonic)	Cardio	Cardio	Cardio
	**Diabetic**	No	No	No	No	Unknown	No	Yes	Yes	Yes
	**Post-surgical days in ICU**	20	0	4	3	2	8	22	4	2
	**Trial date**	15/12/2010	22/11/2010	22/11/2010	24/11/2010	24/11/2010	30/11/2010	30/11/2010	30/11/2010	15/12/2010
**Control details**	**Start time**	17:00	13:10	16:00	14:00	18:00	20:00	17:00	21:00	14:00
	**Initial BG (mg/dL)**	118	159	164	168	223	125	138	127	155
	**Number of nurse interventions that differed from the protocol recommendations**	0	1 intervention of 4 interventions	3 interventions of 9 interventions	7 interventions of 12 interventions	1 intervention of 11 interventions	0	2 interventions of 11 interventions	7 interventions of 11 interventions	2 interventions of 11 interventions
	**Meals**	/	/	/	/	/	/	/	1/12/2011 (9:45): bread (120 mL/h during 15 min)	15/12/2010 (12:00): soup (30 mL/h during 15 min)
									1/12/2011 (12:00): vegetables (not taken into account)	15/12/2010 (19:00): bread, cheese, carrots (90 mL/h during 15 min, maybe underestimated)
									1/12/2011 (18:30): bread (90 mL/h during 15 min)	16/12/2010 (10:00): bread, orange, coffee (90 mL/h during 15 min)
										16/12/2010 (12:00): soup, vegetables, meat (120 mL/h during 15 min)
	**Additional drugs**	/	/	/	Vasopressor (Noradrenaline)	Hydrocortisone	/	/	/	/
	**Vomiting?**	No	No	No	No	No	No	No	No	No
	**Notes**	/	22/11/10 (20:00): trial stopped because patient had big pancreatic illness	/	/	/	/	/	/	/

**Figure 3:**
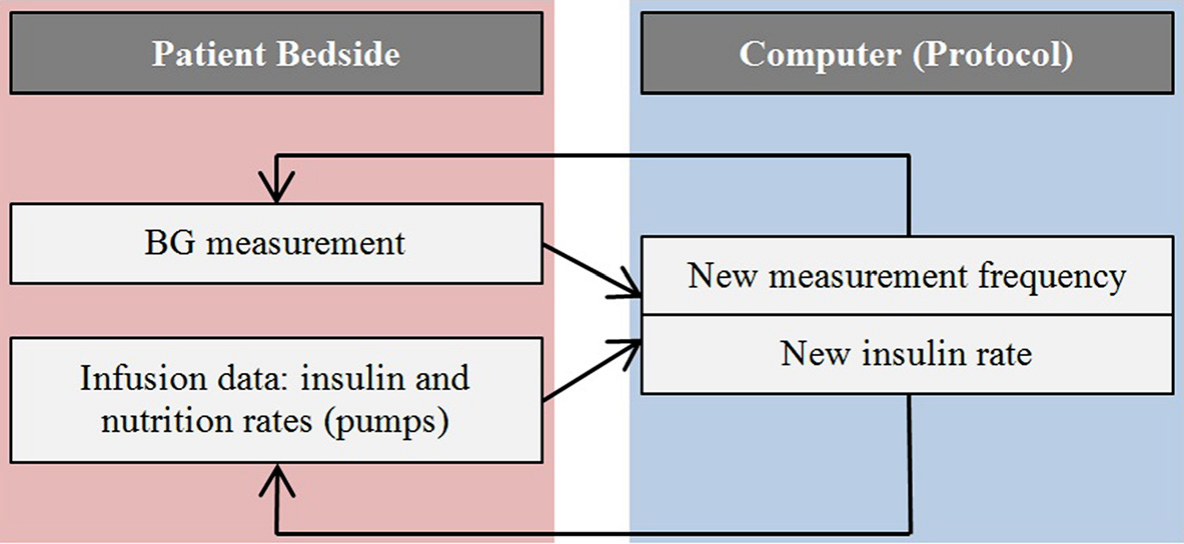
Clinical process during glycemic control.

For each patient, the trial started with a BG measurement made by nursing staff using a bedside glucometer (Accu-Check Inform, Roche Diagnostics, Mannheim, Germany) or a blood gas analyzer (RAPIDPoint 500 Systems, Siemens, Munich, Germany), depending on availability. This measurement was input to the computer and the controller calculated a new insulin infusion rate, which was then used by the nurse on the insulin infusion pumps. This clinical process is illustrated in Figure 3.

### Analyses

Results are compared to the prior and subsequent 24 hours of hospital control to assess performance and safety versus typical hospital control. Results are also assessed compared to SL1 to determine if the goals of reduced workload with no compromise on performance or safety were achieved. In this study, glycemic variability refers to the spread of BG values across the cohort that could be illustrated by the slope of the BG cumulative density function (CDF) or as the interquartile range.

## Results

**Figure 4:**
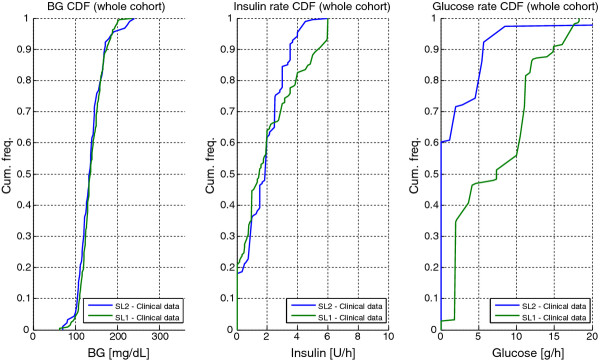
**Whole cohort cumulative density function (CDF) for BG (left panel), insulin rates (middle panel) and exogenous glucose rate (right panel).** Results of the STAR-Liege 1 (SL1, green) and STAR-Liege 2 (SL2, blue) clinical trials are illustrated.

**Figure 5:**
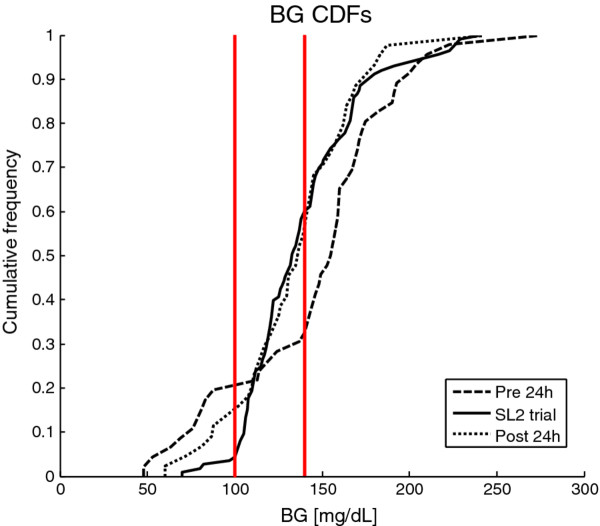
**Cumulative density function (CDF) for BG levels for 24-hour pre-trial, during trial and post-trial for SL2 pilot trial.** Target band bounds (100 mg/dL and 140 mg/dL) are highlighted.

Clinical results are summarized in Tables 2, 3, and 4 and in Figures 4 and 5. A total of 91 measurements were taken over 194 hours, averaging one measurement every 2.1 hours (~11/day). BG levels were relatively tightly distributed, as evidenced by the interquartile range (IQR) range of 33.6 mg/dL (25^th^ – 75^th^ percentile: 117.2 - 150.8 mg/dL) in Table 2 for the cohort and by the IQR for per-patient median values across patients in Table 3. The percentage of BG measurements within the 100–140 mg/dL target band was 54.9% indicating that the control was tight in this band, as illustrated by the steep slope of BG cumulative density function (CDF) for the whole cohort in Figure 4. A total of 40.7% of the BG measurements were ≥ 140 mg/dL primarily due to high initial BG values and short 24-hour trials. The remaining 4.4% of measurements (4 measurements) had BG < 100 mg/dL. There were no severe hypoglycemic events (BG < 40 mg/dL) and the minimum recorded BG was 70 mg/dL (Patient 7). Hence, while STAR forecasted a maximum risk of 5% for BG < 90 mg/dL by design, clinical results show only 3.3%.

**Table 2:** Whole cohort glycemic control results

	**SL2 clinical data**			**SL1 clinical data**	**p-value**
	**Pre 24 hour**	**Pilot trial (+)**	**Post 24 hour**	**Pilot trial (+)**	**SL1 vs. SL2**
Number of patients:	9	9	9	9	/
Total hours:	/	194 h	/	215 h	/
Number of BG measurements:	46	91	44	205	/
BG median [IQR] (mg/dL):	155.5 [124.0 - 171.0]	134.0 [117.2 - 150.8]	136.5 [115.0 - 159.5]	136.0 [122.5 - 158.0]	0.27(*)
% BG within 80–100 mg/dL	8.7	3.3	6.82	3.4	1.00
% BG within 100–140 mg/dL	10.9	54.9	43.18	50.7	0.53
% BG within 140–180 mg/dL	50.0	33.0	36.36	37.1	0.51
% BG ≥ 180 mg/dL	19.6	7.7	9.09	8.3	1.00
% BG < 90 mg/dL	19.6	3.3	11.4	2.0	0.44
% BG < 80 mg/dL	10.9	1.1	4.55	0.5	0.52
% BG < 40 mg/dL	0.0	0.0	0.0	0.0	1.00
Number of patients < 40 mg/dL	0	0	0	0	/
Median insulin rate [IQR] (U/h):	/	2.0 [1.0 - 2.5]	/	1.5 [0.5 - 3.4]	0.92(*)
Median glucose rate [IQR] (g/h):	/	0.0 [0.0 - 5.4]	/	7.4 [2.0 - 11.2]	0.00(*)

The 24-hour pre-trial, during pilot trial and post-trial glycemic data are summarized for SL2 clinical trial. Whole cohort results for SL1 and SL2 pilot trials (+) are compared and p-values were calculated using the Fisher exact test (2-tails) excepted for distributions (*) where a Wilcoxon rank sum test for equal medians was used.

**Table 3:** Per-patient glycemic control results during SL2 pilot trial

	
Initial BG (mg/dL):	155.0 [126.5 - 165.0]
Hours of control:	23.0 [23.0 - 24.0]
Number of BG measurements:	11.0 [10.5 - 11.0]
BG median (mg/dL)	137.0 [120.3 - 142.4]
Median% BG within 80–100 mg/dL:	0.0 [0.0 - 9.1]
Median% BG within 100–140 mg/dL:	54.5 [43.2 - 68.2]
Median% BG within 140–180 mg/dL:	36.4 [18.2 - 46.6]
Median% BG ≥ 180 mg/dL:	0.0 [0.0 - 19.9]
Time to < 125 mg/dL (h):	6.6 [1.9 - 9.0]
% patients to < 125 mg/dL:	88.9
Time to < 140 mg/dL (h):	1.8 [0.0 - 2.6]
% patients to < 140 mg/dL:	100
Median insulin rate (U/h):	1.4 [0.2 - 2.6]
Max insulin rate (U/h):	3.0 [2.9 - 4.0]
Median glucose rate (g/h):	0.0 [0.0 - 4.7]

Statistics are presented as median [IQR] when it’s appropriated.

**Table 4:** Individual patient results during SL2 pilot trial

**Patient**	**Total hours**	**Num. measurements**	**Initial BG (mg/dL):**	**BG median [IQR] (mg/dL):**	**% BG within 80– 100 mg/dL**	**% BG within 100– 140 mg/dL**	**% BG within 140– 180 mg/dL**	**% BG ≥ 180 mg/ dL**	**% BG < 80 mg/ dL**	**% BG < 72 mg/ dL**	**% BG < 40 mg/ dL**	**Min BG level (mg/dL)**	**Median insulin rate [IQR] (U/h):**	**Max insulin rate (U/h):**	**Median glucose rate [IQR] (g/h):**
1	23	11	118	118.0 [112.8 - 135.8]	9.1	72.7	18.2	0	0	0	0	80	0.0 [0.0 - 1.0]	2.0	0.0 [0.0 - 0.0]
2	7	4	159	163.5 [142.5 - 204.0]	0.0	25.0	50.0	25	0	0	0	126	0.3 [0.0 - 2.1]	4.0	4.5 [0.3 - 4.5]
3	23	9	164	137.0 [131.0 - 148.0]	0.0	66.7	33.3	0	0	0	0	122	2.0 [2.0 - 2.5]	3.0	0.0 [0.0 - 0.0]
4	24	12	168	140.5 [130.5 - 146.0]	0.0	50.0	50.0	0	0	0	0	109	2.7 [2.5 - 3.5]	3.5	0.0 [0.0 - 0.0]
5	23	11	223	148.0 [133.8 - 171.0]	0.0	36.4	45.5	18.2	0	0	0	110	4.0 [2.5 - 4.5]	6.0	5.6 [5.6 - 8.5]
6	24	11	125	118.0 [107.0 - 125.8]	9.1	72.7	18.2	0	0	0	0	82	0.0 [0.0 - 1.0]	3.0	0.0 [0.0 - 0.0]
7	23	11	138	138.0 [120.5 - 145.3]	0.0	54.5	36.4	0	9.1	9.1	0	70	2.5 [1.7 - 3.0]	4.0	5.4 [5.4 - 5.4]
8	23	11	127	134.0 [103.8 - 169.0]	9.1	45.5	36.4	9.1	0	0	0	97	1.0 [0.5 - 1.5]	2.5	0.0 [0.0 - 0.0]
9	24	11	155	121.0 [109.0 - 176.8]	0.0	54.5	18.2	27.3	0	0	0	103	1.4 [1.0 - 1.9]	3.0	2.0 [2.0 - 2.0]

For context, BG results are compared to 24-hour pre-trial and 24-hour post-trial BG results of the same nine patients so that each patient acts as their own control. Table 2 shows that SL2 provided better glycemic control compared to the pre-trial period, with 54.9% of BG in the clinically desired band (100–140 mg/dL), instead of 10.9%. This improved control was associated with reduced high BG levels (from 19.6% to 7.7% of BG ≥ 180 mg/dL) and significantly reduced low BG levels (from 10.9% to 1.1% of BG < 80 mg/dL). SL2 did not shift BG levels towards a desired glycemic band, but instead gathered BG levels in a range, as illustrated by the steeper slopes of the BG CDF in Figure 5. The 24 hours following STAR were similar, but more variable, as shown in Figure 5. Overall, STAR successfully reduced BG levels and variability compared to hospital control, while decreasing low BG levels and thus increasing safety.

Table 5 shows details about interventions when nurse interventions differed from protocol recommendations, for insulin rates and/or measurement frequency. Surprisingly, when a 3-hourly option was available, nurses did not always choose this option (2-hourly intervention chosen 11 of 16 cases, 69%, Table 5). Figure 4 shows that no insulin was given in 18% of control interventions, and that insulin rates varied over the full range allowed. Only 5% of insulin rates were higher than 4 U/h, and only Patient 5 received the maximum allowable insulin rate of 6 U/h during the 24-hour trial (Table 4). Nurses overrode 23 (12%) of the 194 interventions recommended by the protocol: 8 (35%) increased insulin rates and 15 (65%) decreased insulin rates (Table 5).

**Table 5:** Details where nurses overrode STAR recommendations during the SL2 pilot trial

		**Protocol recommendations**	**Nurses interventions**	
**Patient 2**	Intervention 1	3.5 U/h for 2 h	1.5 U/h for 1 h	
**Patient 3**	Intervention 1	6 U/h for 3 h	3 U/h for 2 h	-
	Intervention 2	3.5 U/h for 3 h	2.5 U/h for 3 h	*
	Intervention 3	3.5 U/h for 3 h	3 U/h for 3 h	*
**Patient 4**	Intervention 1	4.5 U/h for 2 h	3.5 U/h for 2 h	
	Intervention 2	5.5 U/h for 3 h	3.5 U/h for 2 h	-
	Intervention 3	4 U/h for 2 h	3.5 U/h for 2 h	
	Intervention 4	1 U/h for 3 h or no insulin for 2 h	2 U/h for 2 h	-
	Intervention 5	4.5 U/h for 2 h or 4 U/h for 3 h	3 U/h for 2 h	-
	Intervention 6	2 U/h for 2 h or 3 h or 3 U/h for 3 h	2.5 U/h for 2 h	-
	Intervention 7	4 U/h for 2 h	3.5 U/h for 2 h	
**Patient 5**	Intervention 1	5.5 U/h for 2 h	4.5 U/h for 2 h	
**Patient 7**	Intervention 1	1 U/h for 2 h or 3 h or 1.5 U/h for 3 h	1.5 U/h for 2 h	-
	Intervention 2	No insulin for 2 h or 3 h	1 U/h for 2 h	-
**Patient 8**	Intervention 1	1.5 U/h for 2 h or 3 h	1 U/h for 2 h	-
	Intervention 2	2 U/h for 2 h	1 U/h for 2 h	
	Intervention 3	2 U/h for 3 h or 3 U/h for 2 h	1.5 U/h for 2 h	-
	Intervention 4	No insulin for 2 h or 3 h	0.5 U/H for 2 h	-
	Intervention 5	No insulin for 3 h	0.5 U/H for 3 h	*
	Intervention 6	3.5 U/h for 2 h or 3 h	2.5 U/h for 2 h	-
	Intervention 7	1.5 U/h for 3 h	2 U/h for 3 h	*
**Patient 9**	Intervention 1	No insulin for 3 h	1 U/h for 3 h	*
	Intervention 2	No insulin for 2 h	0.5 U/H for 2 h	

Nurses overrode 23 of 91 interventions. (−): Interventions where nurses chose 2-hourly intervention when 3-hourly intervention is available; (*) interventions where nurses chose 3-hourly intervention when 3-hourly intervention is available.

As mentioned, nutrition input was left to the attending clinician. Approximately 40% of dextrose rates were equal to zero, as five patients received no exogenous dextrose inputs (Patients 3, 4, 6 and 10, Table 4). Clinical results show that the patients were each fed very differently.

Table 2 and Figure 4 show that SL2 achieved somewhat tighter, equally safe control compared to SL1. BG levels were similarly distributed (p > 0.05), while the number of measurements was reduced by 55.6% (p < 0.05). SL2 had slightly lower insulin rates due to the significantly lower exogenous glucose administration rates (p < 0.01).

## Discussion

The SL2 protocol was primarily designed to reduce nurse workload, while maintaining safety and control. Three main changes were made. First, while SL1 was characterized by a specific glycemic target of 125 mg/dL, SL2 used a target-to-range approach (target band: 100–140 mg/dL). Second, measurement frequency was reduced as only 2-hourly and 3-hourly interventions were used, instead of the 1- and 2- hourly interventions during the first trial. Third, the SL2 protocol had fewer rules (for example, it did not adjust nutrition rates), which made the protocol more simple and transparent, and its application faster. Additionally, the controller used an improved model of the glucose-insulin system [19] and a cohort-specific stochastic model to account for a more variable cardiovascular cohort [13].

Nurse workload was significantly reduced with the SL2 protocol (2.1 hours between measurements vs. 1.1 for SL1, p < 0.01). Table 5 shows that nurses sometimes choose 2-hourly interventions (31% of time) when a 3-hourly option was available. This result indicates that measurement frequency could have been further reduced if nurses chose 3-hourly interventions when available. Hence, nurse workload could have been further reduced.

Nurses overrode insulin rates more often during the SL2 clinical trial than during the SL1 clinical trial. This difference can be explained by some “lack of trust” in the recommendations, especially as the time interval was longer. Nurses were hesitant to administer more than 3 U/h, and were quite resistant to insulin rate changes (Table 5). However, 35% of override changes increased insulin over recommendations. Table 2 and Figure 5 show that hospital control was less effective and more variable than STAR, so this non-compliance may not have improved control.

SL2 explicitly defined a maximum hypoglycemic risk of 5% of BG < 90 mg/dL. In contrast, SL1 used a maximum 5% risk of BG < 72 mg/dL [18]. During the SL1 trial, there were 2.0% of BG < 90 mg/dL, representing 4 of 205 BG measurements. During the SL2 trial, there were 3 of 91 BG < 90 mg/dL (3.3%). This percentage (and number) of BG < 90 mg/dL are acceptable as it is less than the desired maximum of 5% (~ 4–5 BG measurements over 91). Despite less frequent measurement and intervention, safety was still ensured, and matched design levels.

The relatively short length of each trial does not allow long-term statistics on control. However, a median 1.8 hours to BG < 140 mg/dL indicates total trial length was sufficient to test safety and efficacy compared to SL1. The results justify longer trials for 48 or more hours.

A main difference between the SL1 and SL2 results was the reduced intervention rate, which can increase BG variability in patients whose condition changes rapidly. However, the longer intervals allowed the effect of changes in insulin infusion rate to be more clearly observed and identified, compared to bolus administration in other uses [21] which act more quickly and can thus be more rapidly identified. However, these results indicate no increase in variability or risk as a result.

Some situations are still not automatically managed by STAR. In particular, small meals may be given (Patients 8 and 9, Table 1) which are difficult to estimate. The added estimated exogenous glucose content was included in control. However, incomplete consumption and estimated exogenous glucose content adds uncertainty, although STAR appeared to manage this issue as well as, or better, than normal hospital control. Future efforts need to include this aspect more explicitly.

Finally, this clinical trial includes only 9 subjects. Longer trials over more patients would provide greater certainty to the results. However, it is clear that the goals of reducing workload without compromising safety or performance were met. Equally, it is clear that STAR was better than the normal hospital protocol. The STAR protocol gathered BG levels around the desired glycemic band, reduced high BG levels and improved safety by significantly reducing low BG levels. STAR also positively impacted on 24-hour post-trial glycemic results. Hence, STAR also helped stabilizing patient condition and helped further patient management.

## Conclusion

The main objective for these second clinical trials was to reduce clinical workload, while maintaining control quality and safety, using a target-to-range approach. Results show that clinical workload was reduced by over a factor of 2, while safety was maintained with less frequent measurement and intervention compared to prior clinical trial. The results presented thus show that safe, effective glycemic control can be achieved for a highly variable cohort with significantly reduced workload using a model-based method, where several clinical studies on similar cardiovascular cohorts have had excessive hypoglycemia. Moreover, STAR was shown to be safer and tighter than the existing hospital control.

## Abbreviations

BG: Blood glucose; CDF: Cumulative density function; CHU: Centre hospitalier universitaire; ICU: Intensive care unit; IQR: Interquartile range; SI: Insulin sensitivity; SL1: First STAR-Liege (trial); SL2: Second STAR-Liege (trial); SPRINT: Specialized relative insulin and nutrition tables; STAR: Stochastic targeted; TGC: Tight glycemic control.

## Competing interests

The authors declare that they have no competing interests.

## Authors’ contribution

ALC developed the SL2 model-based protocol. SP and KTM conducted the trial and made the acquisition of data during the clinical trials. All authors were involved in the analysis and interpretation of data. The manuscript was originally drafted by SP, and JGC, but all authors made contributions through the entire process, including reading and final approval of this manuscript.

## Authors' information

MSc, Cardiovascular Research Centre, Institut de Physique, Université de Liege, Institut de Physics, Allée du 6 Août, 17 (Bât B5), B4000 Liege, Liege, Belgium.

PhD, Cardiovascular Research Centre, Institut de Physique, Université de Liege, Institut de Physics, Allée du 6 Août, 17 (Bât B5), B4000 Liege, Liege, Belgium.

PhD, Department of Mechanical Engineering, Centre for Bio-Engineering, University of Canterbury, Christchurch, Private Bag 4800, 8054, New Zealand.

MSc, Department of Mechanical Engineering, Centre for Bio-Engineering, University of Canterbury, Christchurch, Private Bag 4800, 8054, New Zealand.

PhD, Department of Mechanical Engineering, Centre for Bio-Engineering, University of Canterbury, Christchurch, Private Bag 4800, 8054, New Zealand.

MD, PhD, Department of Intensive Care, Erasme University Hospital, 808 route de Lennik, B1070 Brussels, Belgium.

MB, ChB, Department of Intensive Care, Christchurch Hospital, Christchurch, 8054, New Zealand

MD, PhD, Department of Intensive Care, CHU, Liege, Belgium.

## Supplementary Material

Additional file 1 This file provides the description of the glucose-insulin model. Click here for file

Additional file 2 This file provides the description of the stochastic model. Click here for file
