# Supplementary material for: Second pilot trials of the STAR-Liege protocol for tight glycemic control in critically ill patients
Source: Biomed Eng Online. 2012 Aug 23;11:58. doi: 10.1186/1475-925X-11-58 (PMC3511234; doi:10.1186/1475-925X-11-58)
Supplement: Additional file 1 — This file provides the description of the glucose-insulin model. [file 1475-925X-11-58-S1.docx]

A-1: Definition of the glucose-insulin system model.

Equations (1) to (5) define the clinically validated model (named ICING 2, [[19](#_ENREF_19)]) of the glucose-insulin system that describes the evolution of 5 variables: the blood glucose level ($G$), the plasma and interstitial insulin concentrations ($I$ and$Q$, respectively), the glucose concentration in the stomach and in the gut ($P_{1}$ and$P_{2}$, respectively).

| $\dot{G}=-p_{G}.G-S_{I}.G.\frac{Q}{1+\alpha_{G}.Q}+\frac{\min(d_{2}.P_{2},P_{max})}{V_{G}}+ \frac{EGP}{V_{G}}+ \frac{PN}{V_{G}}- \frac{CNS}{V_{G}}$ | (1) |
| --- | --- |
| $\dot{I}=-I.n_{K}-n_{L}.\frac{I}{1+\alpha_{I}.I}-\left( I-Q \right).n_{I}+\frac{u_{ex}}{V_{I}}+\frac{\left( 1-x_{L} \right).u_{en}}{V_{I}}$ | (2) |
| $\dot{Q}=\left( I-Q \right).n_{I}- n_{C}.\frac{Q}{1+\alpha_{G}.Q}$ | (3) |
| $\dot{P_{1}}=-d_{1}.P_{1}+P$ | (4) |
| $\dot{P_{2}}=-\min(d_{2}.P_{2},P_{max})+d_{1}.P_{1}$ | (5) |

Where $u_{ex}$is the exogenous insulin input, $u_{en}$ is the endogenous insulin production, $P$ is the exogenous enteral dextrose input and $PN$ is the exogenous parenteral dextrose input. Table A summarizes the model parameter definition.

| $p_{G}$ | Non-insulin mediated glucose removal parameter | 0.006 | [1/min] |
| --- | --- | --- | --- |
| $CNS$ | Central nervous system glucose uptake | 0.3 | ?? |
| $EGP$ | Endogenous glucose production | 1.16 | [mmol/min] |
| $x_{L}$ | First-pass liver extraction of insulin | 0.67 | [] |
| $n_{I}$ | Diffusion rate between $I$ and $Q$ | 0.0075 | [1/min] |
| $n_{K}$ | Kidney insulin clearance | 0.0542 | [1/min] |
| $n_{L}$ | Liver insulin clearance base rate | 0.1578 | [1/min] |
| $n_{C}$ ($=n_{I})$ | Interstitial insulin degradation base rate | 0.0075 | [1/min] |
| $d_{1}$ | Glucose transfer rate from stomach to gut | -log(0.5)/20 | [1/min] |
| $d_{2}$ | Glucose transfer clearance rate from gut | -log(0.5)/100 | [1/min] |
| $V_{I}$ | Plasma insulin distribution volume | 4.0 | [L] |
| $V_{G}$ | Plasma glucose distribution volume | 13.3 | [L] |
| $V_{X}$ | Subcutaneous insulin distribution volume | 0.1421*80 | [L] |
| $\alpha_{I}$ | Insulin clearance saturation parameter | 1.7e-3 | [L/mU] |
| $\alpha_{G}$ | Insulin binding saturation parameter | 1/65 | [L/mU] |
| $P_{max}$ | Maximum glucose flux out of the gut | 6.11 | [mmol/min] |

Table A – Model parameter definition.
