# Supplementary material for: Second pilot trials of the STAR-Liege protocol for tight glycemic control in critically ill patients
Source: Biomed Eng Online. 2012 Aug 23;11:58. doi: 10.1186/1475-925X-11-58 (PMC3511234; doi:10.1186/1475-925X-11-58)
Supplement: Additional file 2 — This file provides the description of the stochastic model. [file 1475-925X-11-58-S2.docx]

A-2: The stochastic model for insulin sensitivity.

The insulin sensitivity is the critical model-based parameter in predicting the outcome of an insulin intervention. As insulin sensitivity is relatively variable hourly, modeling the changes in insulin sensitivity is quite important to improve assessment of the patient’s insulin response, and thus to allow more accurately targeted control. The insulin sensitivity variability accounts for the adaptation of the entire model to the patient condition during the control.

The goal of the stochastic model is to describe the hourly variations of the insulin sensitivity, based on clinically observed insulin sensitivity variations in ICU population data. First, clinical data (blood glucose, insulin and nutrition inputs) are used to identify hourly insulin sensitivity values using the glucose-insulin system model (A-1) [[20](#_ENREF_20)]. If insulin sensitivity at hour n+1 and at hour n are denoted respectively by $S_{I,n+1}$ and $S_{I,n}$, the distribution of the hourly changes in $S_{I}$, ($S_{I,n}=x_{i},S_{I,n+1}=y_{i}$), can be assessed (Figure A, left panel). Finally, a probability density of insulin sensitivity at hour n+1 ($S_{I,n+1}$) taking on a value $y$ can be calculated by knowing insulin sensitivity at hour n ($S_{I,n}$), using identified variations from clinical data. This probability density function,$p(S_{I,n+1}=y|S_{I,n}=x)$, defined the stochastic model. An example is illustrated on the right panel in Figure A.


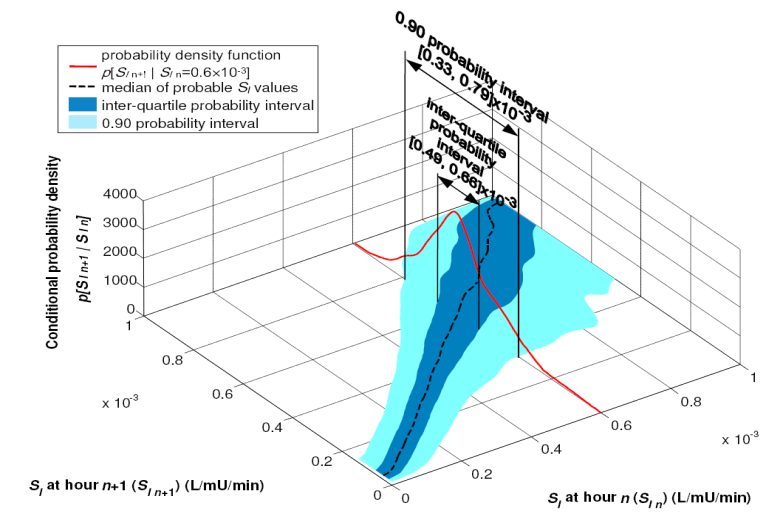


Figure A – Left panel: distribution of hourly changes in SI. Right panel: SI probability density function.

The stochastic insulin model can then be used to forecast likely blood glucose outcomes for a given intervention (a given insulin infusion), using the model defined by Equations (1) to (3). This approach, illustrated in Figure B, allows the optimization of prediction and ensures safety, especially from hypoglycemia.

Stochastic model shows the bounds (5^th^, 25^th^, 50^th^=median, 75^th^ and 95^th^ percentiles) for insulin sensitivity variation over the next 1-2 hours from initially identified level.

For a given insulin intervention, an output BG distribution can be forecast using the model of glucose-insulin system.

$$t_{now}$$

$t_{now}$+(2-3) h

Figure B – Forecasted BG outcomes.
